# Epilepsy and Employment in Europe: A Systematic Review of Literature

**DOI:** 10.1111/ene.70129

**Published:** 2025-04-21

**Authors:** F. Narducci, G. Lippa, G. Baker, D. Walsh, F. Sofia, N. Casalino, F. Pigni, S. Louissi, I. Philipova, S. Duttenhöfer, L. Ricci, B. Sancetta, J. Lanzone, M. Tombini, V. Di Lazzaro, G. Assenza

**Affiliations:** ^1^ Research Unit of Neurology, Department of Medicine and Surgery Università Campus Bio‐Medico di Roma Rome Italy; ^2^ Operative Research Unit of Neurology Fondazione Policlinico Universitario Campus Bio‐Medico Rome Italy; ^3^ Department of Molecular and Clinical Pharmacology University of Liverpool Liverpool UK; ^4^ International Bureau of Epilepsy (IBE) Dublin Ireland; ^5^ Department of Business and Management Luiss Guido Carli University Rome Italy; ^6^ Management and Technology, Grenoble Ecole de Management Grenoble France; ^7^ Chamber of Commerce and Industry Vratsa Bulgaria; ^8^ Emcra GmbH Berlin Germany; ^9^ Neurology Unit and Neurophysiology Service IRCCS San Raffaele Scientific Institute Milan Italy

**Keywords:** attitudes, disclosure, employment, epilepsy, seizure severity, stigma, underemployment

## Abstract

**Background:**

Epilepsy is a chronic disorder affecting all aspects of individual life. People with epilepsy (PwE) reach seizure control in about 60% of cases. However, social integration issues are frequently overlooked. Unemployment and underemployment are markedly more common in PwE compared with the general population. With this review, we aimed to depict the current state of the employment situation in Europe with a focus on factors that may influence it.

**Methods:**

We performed a systematic review on epilepsy and employment as part of EpilepsyPOWER Erasmus+ project (2021‐1‐IT02‐KA220‐ADU‐000028349). Our search string was “Epilepsy AND Employment OR Job OR Work.” Using the Preferred Reporting Items for Systematic Reviews and Meta‐Analyses (PRISMA 2020) guidelines, we screened 7272 articles and selected 55 articles from 1958 to 2023. We extrapolated data on employment rate and status, also considering people with specific epileptic syndromes. We finally evaluated factors contributing to employment and unemployment.

**Results:**

Unemployment rates range from similar to twice or three times the rates of the general population, depending on the countries and years examined. When analyzing factors contributing to employment conditions, most papers highlighted the importance of seizure control and employers' attitudes.

**Conclusion:**

Developing specific legislation and programs to include PwE in the workplace could help their social integration. Moreover, seizure control seems to be the most relevant factor influencing the possibility of getting and maintaining a good job, demonstrating the importance of providing continuous follow‐up and the best medical care to all PwE.

## 1 Introduction

Epilepsy is a common neurological disorder, affecting all aspects of the individual's life, including social integration. Seizures represent only a small piece of the complex and heterogeneous picture of this disorder. Drugs' side effects, psychiatric comorbidities, cognitive deficits, and driving limitations increase the burden of epilepsy and negatively impact educational attainment and employment [1, 2].

False beliefs, misconceptions, and employers concerns contribute to unemployment in PwE [3, 4, 5]. Thus, we can describe the employment situation in PwE as a combination of internal (seizures, cognitive deficits, etc.) and external factors (stigma, employers' attitudes, etc.).

Employment is one of the main determinants of economic independence, self‐worth, and individual identity. Being unemployed prompts a lack of independence, reduced self‐esteem, increased feelings of stigma, and consequently, a lower quality of life [6, 7].

Despite good seizure control, unemployment often affects PwE [2]. The lack of knowledge, specific legislation, and inclusion programs hamper employment integration. Three key pieces of legislation (exceptions) worldwide must be highlighted: the Americans with Disabilities Act (ADA) in the United States, the Health and Safety at Work Act (1974), and the Equality Act (2010) in Europe, which covers epilepsy among general disability.

When hiring PwE, employers' misconceptions often include fear of higher absenteeism and accidents. Conversely, it has been shown that the risk of seizure‐related accidents is comparable to that of injury not related to seizures [8]. Some studies found a higher risk of accidents among PwE, but we might notice that they were not related to seizures. Even though absenteeism and accidents are not higher in employees with epilepsy, PwE salaries appear to be lower than those of their colleagues [9].

Although many authors attempted to describe the employment situation worldwide [1, 10, 11], estimating the real unemployment rate is still challenging. Epilepsy can be a “hidden condition”: PwE can conceal their disorder from employers because of fear or stigma. This scenario can be interpreted as a break of trust [5]; thus, another key issue for employment among PwE is disclosure.

Aiming to obtain the maximum grade of scientific evidence from the available specific literature, we conducted a systematic review on the employment rate of PwE and factors influencing their employment and unemployment in Europe.

We focused on European literature as this review is a key activity of the EpilepsyPOWER project (2021‐1‐IT02‐KA220‐ADU‐000028349), financed by the European Commission and Erasmus+, involving five European countries (Italy, Ireland, Bulgaria, France, Germany). The objectives of the EpilepsyPOWER project aim to improve the inclusion of PwE in the job market, increase the number of people engaged in relevant corporate social responsibility epilepsy‐friendly initiatives, spread culture and practice for the implementation of inclusion systems for PwE, and support universities, companies, and small enterprises in inclusion improvement.

## 2 Methods

### 2.1 Research Strategy

We sought articles about epilepsy and employment on PubMed, Google Scholar, and Embase.

Our search string was “Epilepsy AND Employment OR Job OR Work”. We used the Rayyan review system, an online tool allowing for flawless collaborative systematic review [12].

Using the Preferred Reporting Items for Systematic Reviews and Meta‐Analyses (PRISMA 2020) guidelines, we performed a systematic review on epilepsy and employment [13].

**FIGURE 1:**
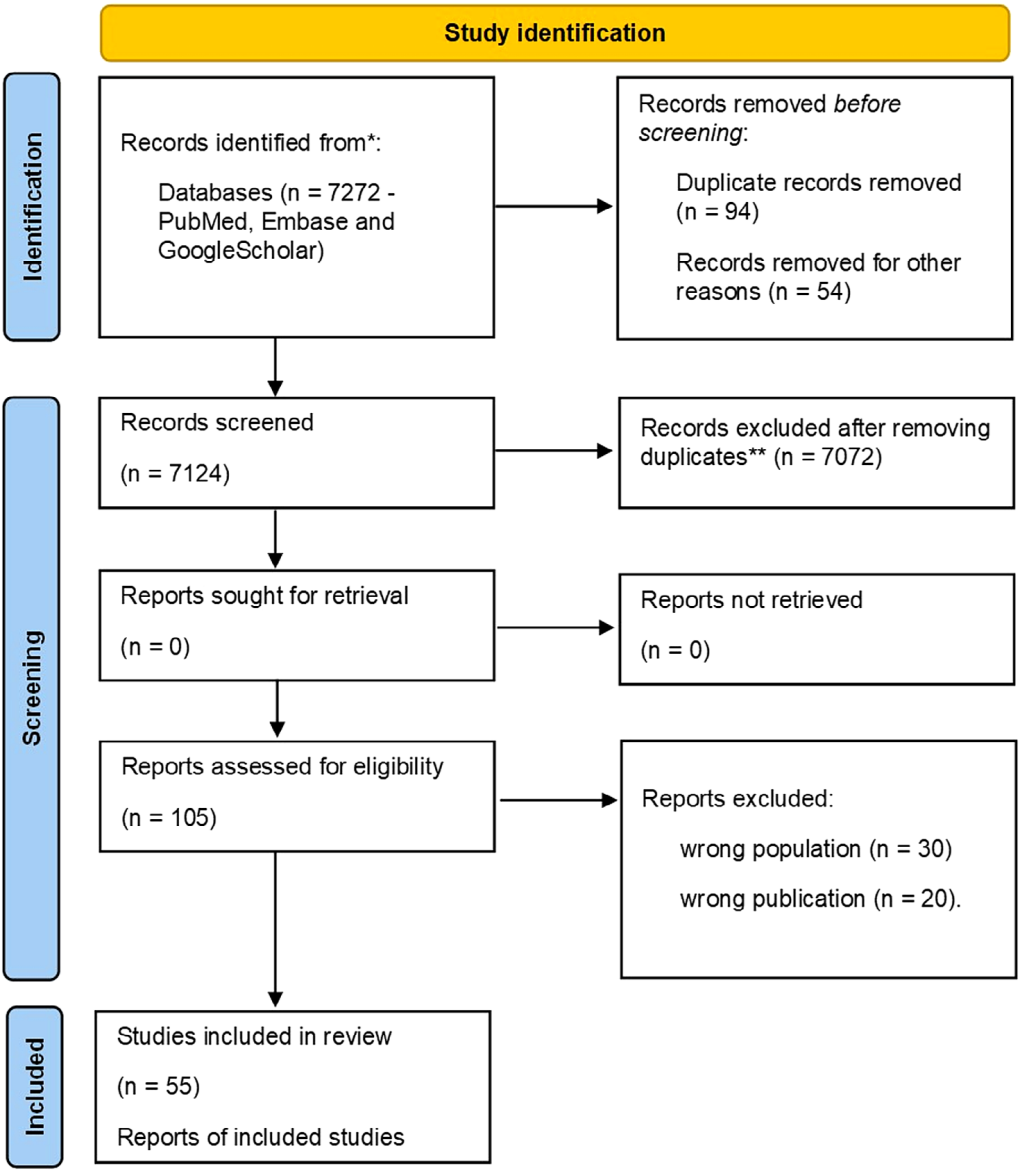
Preferred reporting items for systematic reviews and meta‐Analyses method.

We selected 55 among 7272 articles from 1958 to 2023 (Figure 1).

We then grouped those articles between papers reporting employment status and papers only describing factors contributing to employment/unemployment. We also considered specific cases: surgical patients and people with specific syndromes (e.g., juvenile myoclonic epilepsy, childhood‐onset epilepsy, absence epilepsy).

### 2.2 Inclusion and Exclusion Criteria

We have only included articles written in English and published in journals with good impact factors (IF) regarding employment conditions in different European countries, as a specific deliverable of the European Project EpilepsyPOWER (2021‐1‐IT02‐KA220‐ADU‐000028349). We considered articles regarding people living in the UK, as it was part of the geographic area of the European Union (EU) and politically included in the EU until 2023. We excluded articles with wrong types of publication (e.g., letters to editors) or wrong populations (e.g., people with disabilities and not suffering from epilepsy).

### 2.3 Study Selection

We screened papers using the artificial intelligence automatic Rayyan review system [12]. Abstracts were selected by two investigators (GL and the Principal investigator, FN), based on titles and abstracts. Two researchers independently reviewed selected articles and results. GA supervised the quality of selected papers and adherence to the proposed aim, trying to minimize the risk of selection biases.

We selected all papers containing the world “employment” and/or “employed” in the title or in the abstract, dividing them into manuscripts reporting rates of employment, articles reporting specific situations (including surgical outcomes), and disclosure issues.

### 2.4 Employment Status

Selected manuscripts considered different sample dimensions, and they often did not report the specific rate of employment/unemployment.

Only a few articles (eight) specified rates of retired PwE; the others included them as unemployed people, likewise for housewives and people receiving disability pensions. No article specifically mentioned self‐employment, and only three papers mentioned part‐time jobs. Therefore, despite the limitations, this category was considered among employed PwE.

Therefore, due to the above limitations, the evaluation of the real unemployment rate was not completely clear. Thus, we decided to list articles reporting the same/different rates of employment/unemployment compared with the general population, also to find out a trend in recent years. We specified the type of study, rates of employment/unemployment reported, and comparison with the general population.

## 3 Results

Among manuscripts selected:

- Forty‐one manuscripts mentioned employment status: nine manuscripts reporting similar rates of employment/unemployment compared with the general population; four manuscripts reporting different rates of employment/unemployment compared with the general population (Tables 1 and 2).
- Eight manuscripts focused on surgical outcomes;
- Forty‐five manuscripts reported factors contributing to employment/unemployment;
- Four manuscripts regarded specific subtypes of epilepsy: childhood‐onset epilepsy, absence epilepsy, juvenile myoclonic epilepsy, and medial temporal lobe epilepsy;
- Six manuscripts mentioned disclosure issues.

**TABLE 1:** Papers reporting similar rates of employment/unemployment.

Title	Author	Year	Country	Type of study	Employment status	Population (N)	Comparison with general population
**Similar rates**							
Social class, epileptic activity, and disadvantage at work	Scambler	1980	UK	Survey	42/73 of people of working age employed in full work 31/42 men (74% vs. 81% in general population) 6/19 married women (32% vs. 48% in general population) 5/12 unmarried women (42% vs. 42% in general population)	94 PwE (73 respondents)	Similar rate
Epilepsy and the quality of everyday life. Findings from a study of people with well‐controlled epilepsy	Jacoby	1992	UK	Survey	79% of men and 63% of women under 60, employed	549 PwE	Similar rate
Correlates of employment history and employability in a British epilepsy sample	Collings	1994	UK	Survey	58% employed 12% unemployed (vs 9% in general population) 18% students/housewife other	1709 PwE	Similar rate
Impact of epilepsy on employment status: Findings from a UK study of people with well‐controlled epilepsy	Jacoby	1995	UK	Prospective randomized controlled trial	71% employed 26% unemployed because of other reasons 3% unemployed because of seizures	607 PwE	Similar rate
Felt stigma and impact of epilepsy on employment status among Estonian people: exploratory study	Rätseep	2000	Estonia	Survey	Full time employed 38.9% Part time employed 24.0% Unemployed 11% Retired 10% Receiving disablement pension 15.6%	90 PwE	Similar rate
Epilepsy in Estonia: A Quality‐of‐Life Study	Herodes	2001	Estonia	Survey	33% full time employed; 31.9% underemployed; 11%, unemployed (vs 9.5% in general population); 24.1% retired/disability pension	203 PwE	Similar rate
Health perception and socioeconomic status following childhood‐onset epilepsy: The Dutch study of epilepsy in childhood	Geerts	2011	Netherlands	Prospective cohort study	A total of 139 subjects (33.7%) had a job Employment rates: idiopathic 91.5%, symptomatic 91.3%, cryptogenic 97.1%, in remission 93.2%, active epilepsy 91.2%, Dutch age peers 92.0%.	413 people with childhood‐onset epilepsy	Similar rate
Socio‐occupational and employment profile of patients with epilepsy	Marinas	2011	Spain	Cross‐sectional multicenter epidemiological study	Employed PwE: 504 (58%) compared with 59.35% in first trimester of 2008 reported by Spanish National Statistics Institute Unemployed PwE95 (10.9%) Student 49 (5.6%) Occupational incapacitation 109 (12.5%) Housewife 107 (12.3%) Employed and studying 5 (0.6%)	872 adult PwE	Similar rate
Long‐term employment outcomes after epilepsy surgery in childhood	Reinholdson	2019	Sweden	Population based study	At the 10‐, 15‐, and 20‐year follow‐ups, 17/25 (68%); 22/29 (76%); 8/13 (62%) of the seizure‐free patients aged ≥ 25 years worked full time. In the general population, the corresponding weighted figures were 66%, 66%, and 68%	203 surgical patients	Similar rate

**TABLE 2:** Papers reporting different rates of employment/unemployment.

Title	Author	Year	Country	Type of study	Employment status	Population (N)	Comparison with general population
**Different rates**							
Epilepsy and employment, marital, education and social status	Callaghan	1992	Ireland	Survey	34% of males and 28% of females unemployed (compared with 13% of males in general population); 53% of males and 24% of females gainfully employed; 1% of males and 41% of females house duties; 8% of males and 7% of females in sheltered employment; 4% of males and < 1% of females retired	343 PwE	Higher rate of unemployment
Epilepsy‐related employment prevalence and retirement incidence in German working population: 1994–2009	Korchounov	2012	Germany	Longitudinal study	Mean employment rate of PwE was lower than that of the general population (68.5% vs. 90.1%, *p* < 0.001)	n/a	Lower rate of employment
Employment in people with epilepsy from the perspectives of patients, neurologist and the general population	Majkowska‐Zwolinska	2012	Poland	Survey	486 (49%) PwE were professionally active (compared with 59.3% in Poland and 64.6% in Europe), 508 (51%) PwE were unemployed	995 PwE (18–65 years), 179 neurologists, and a representative sample of the Polish population over 15 years of age (1042)	Lower rate of employment
Prospective and longitudinal long‐term employment outcomes after resective epilepsy surgery	Edelvik	2015	Sweden	Longitudinal study	Full‐time work 33.5% at 2 years; 35.5% at 5 years; 35.0% at 10 years; 32.1% at 15 years. Part‐time work 17.9%; 17.1%; 17.7% 25.0%. In the general population 65%–71% (25–54 years old) worked full‐time Student 11.3%; 11.1%; 11.8%; 6.0% Benefits/unemployed 35.3%; 34.1%; 32.5%; 34.5% (rates reported were at 2–5‐10 years of FUP).	473 PwE (85 complete FUP)	Lower rate of employment

### 3.1 Employment Status and Unemployment Rate

Unemployment rates reported among PwE were different across Europe.

Most of the articles came from the UK (12), whereas other articles examined the situation of the Netherlands (6), Germany (5), Sweden (4), Estonia (3), Spain (3) Ireland (2), Denmark (2), Poland (2), and Finland (2).

Here, we list articles comparing rates of employment/unemployment with the general population, in chronological order (Tables 1 and 2).

Scambler in 1980, interviewing general practitioners, described the following employment situation in the UK: 31/42 (74%) of men of working age, 6/19 (32%) of married women, and 5/12 (42%) of unmarried women were employed, with respective rates in the general population of 81% (men), 48% (married women), and 42% (unmarried women) [14].

Several studies conducted by Ann Jacoby analyzed the employment situation in the UK in the 90s, both from the employers and employees' perspective [5, 15, 16]. Among those studies, in 1992 and 1995, she demonstrated a similar rate of employment in people with well‐controlled epilepsy compared with the general population. In 1992, rates reported among PwE were 91% of men and 74% of women under 60 employed; of those unemployed, only 3% reported epilepsy as the main unemployment reason [15]. Similar results were described in 1995: among 494 PwE of working age, 71% were employed, 26% unemployed, and 3% of those unemployed reported seizures as the reason for unemployment [16]. Collings reported a rate of unemployment of 12% compared with the 9% national unemployment rate in the UK in 1994 [4].

In 1992, Callaghan described the employment situation in Ireland in a population of 343 PwE: 34% of males and 28% of females were unemployed; 53% of males and 24% of females were gainfully employed; 1% of males and 41% of females did house duties; 8% of males and 7% of females were involved in sheltered employment; and 4% of males and less than 1% of females were retired. They also highlighted higher underemployment among PwE (34%) compared with the general population (13%) in the same years [17].

On the contrary, Kourchonov evidenced a lower rate of employment in PwE in Germany, despite the promulgation of the Law on Support of Employment in 1996 [18].

In more recent years, Herodes and Rätsepp both demonstrated similar rates of unemployment compared with the general population in Estonia [19, 20].

Geerts reported similar rates of employment among people with childhood‐onset epilepsy compared with the general Dutch population in a longitudinal study, regardless of the specific kind of epilepsy: idiopathic epilepsy 91.5%, symptomatic epilepsy 91.3%, cryptogenic epilepsy 97.1%, epilepsy in remission 93.2%, active epilepsy 91.2%, all compared with Dutch age peers 92% [21].

A cross‐sectional multicentric epidemiological study in Spain also showed similar rates of employment and unemployment in PwE compared with the general population [22].

Recently in Poland, Majkowska demonstrated lower rates of employment in PwE (49%) compared with the Polish and European populations (59.3% and 64.6%, respectively) [23].

As for surgical patients, Eldevik compared the employment rate of seizure‐free patients after surgery in a prospective longitudinal study. In the Swedish general population, 65%–71% of those between 25 and 54 worked full time, compared with 36% of seizure‐free patients 5 years after surgery and 65% 10 years after surgery [24].

Considering surgical treatment outcomes, Reinholdson showed that seizure‐free PwE with intelligence quotient IQ > 70 gain the same level of employment as the general population [25].

In the last decades (2000–2023), a slight prevalence of articles reporting similar rates of employment (five vs. three) has emerged.

### 3.2 Factors Contributing to Unemployment and Underemployment

Unemployment and underemployment in PwE derive from a combination of multiple and different factors. Internal factors include individual characteristics, clinical features, and self‐concepts. Whereas, work atmosphere, knowledge, and employers' attitudes represent the main external determinants of employment status in epilepsy (Table 3) [10].

**TABLE 3:** Most relevant internal and external factors contributing to employment and unemployment among PwE (mentioned in at least three articles).

Factors	Employment	Unemployment
**Individual factors**	**Seizure control and remission** (Sorel 1972; McLellan 1987; Collings 1994; Jacoby 1995; Jacoby 1996; Chaplin 1998; The RESt‐1 Group 2000; Dupont 2006; Sillanpaa 2010; Jennum 2011; Schulz 2013; Majkowska‐Zwolinska 2012; Edelvik 2015; Walther 2018) **Ongoing treatment with lower number of antiseizure medications** (DeBoer 2005; Haag 2010; Jennum 2011; Marinas 2011; Korchounov 2012; Schneider‐Von Podewils 2014) **Surgery** (Dupont 2006; Carreño 2008; Walther 2018) **Normal IQ and cognitive function** (Sillanpaa 2010; Reinholdson 2019; Partanen 2022)	**Early onset** (Pond 1960; Herodes 2001; Jacoby 2005; Geerts 2011; Walther 2018; Jennum 2020) **Seizure severity, type & frequency** (Scambler 1980; Jacoby 1992; Callaghan 1992; Jacoby 1995; Jacoby 1996; Ratseep 2000; Herodes 2001; De Boer 2005; Jacoby 2005; Koponen 2007; Marinas 2011; Schneider‐von Podewils 2014; Walther 2018; Reinholdson 2019; Mireia 2021) **Cognitive deficits** (Gordon & Russel 1958; Thompson 1988; Geerlings 2015) **Psychiatric comorbidities** (Pond 1960; Gloag 1985; Jacoby 1996; Peña 2008; Jennum 2011; Mireia 2021)
**External** **factors**	**Training and job counseling** (Sorel 1972; Carroll 1992; Chaplin 1998) **Higher education** (Collings 1994; Ratseep 2000; Herodes 2001; Koponen 2007; Majkowska‐Zwolinska 2012; Edelvik 2015) **Favorable atmosphere at work and positive individual perception** (Graham 1965; Collings 1994; Jacoby 2005)	**Stigma and misconceptions** (Scambler 1980; Chaplin 1992; Chaplin 1998; Jacoby 2005) **Lower education** (McLellan 1987; Buchanan 1988; Callaghan 1992; Geerts 2011; Marinas 2011; Mireia 2021) **Employers' attitudes and unsupportive familial environment** (Gloag 1985; Elwes 2015; Thompson 1988; Geerlings 2015)

Among individual factors, younger age and early onset of disease seem to negatively affect the chance of obtaining a good job position [19, 26, 27]. Indeed, in a Finnish study, Sillanpaa evidenced that onset at age less than 6 is one factor promoting employment in PwE [28]. According to Geerts, childhood‐onset epilepsy is strictly connected to lower educational success, and, so, to a lower job attainment [21]. On the contrary, Haag found that older people had a lower probability of being employed. Jacoby demonstrated comparable results in a large cohort study in the UK in 1996 [29].

When considering clinical factors, the most important determinants of employment are seizure freedom and remission. Multiple studies indicated seizure severity as the main explanation for unemployment in PwE: in 1992, Jacoby, focusing on a population of well‐controlled PwE, stated that active epilepsy (seizures occurring in the last 2 years) impaired employment conditions [15]. Three years later, the same group established in a similar population that seizure severity, frequency, and controllability negatively affect employment in PwE [16]. Refractory epilepsy may hamper long‐term social outcomes, including employment [22]. Conversely, seizure remission and having no history of status epilepticus are positive factors in people with childhood‐onset epilepsy [28]. Later, other authors asserted that a higher seizure frequency was linked to unemployment [14, 16, 20, 25, 30, 31, 32]. When considering long‐term outcomes, treatment with antiseizure medications prompts seizure control and, in most cases, seizure freedom: thus, it can be considered as another clinical factor connected to employment [33, 34]. Seizure type can also influence employment: tonic–clonic seizures are linked to a worse employment status [16, 34].

Another relevant element is cognition: cognitive deficit and intellectual constraints consistently impair job attainment [32, 35, 36]. Academic difficulties and lower educational levels also contribute to unemployment [37, 38]. Social difficulties [36] and psychiatric comorbidities [33, 38, 39], anxiety and depression [16, 37, 38, 40] might also impair employment. In general, comorbid disabilities lead to lower educational levels and chances of employment, as demonstrated in a population of surgical patients in Germany [41]. Polytherapy also affects employment status and quality of life [22, 42]. Clearly, a higher number of drugs can affect cognitive capacities and concentration, and so the ability to work normally.

Stigma, misconceptions, and misbeliefs surrounding epilepsy lead to discrimination from employers and self‐denial of employees in the workplace [14, 30, 36]. Employers' attitudes and an unsupportive familial environment can hamper gaining a good job position [5, 32, 36, 43]. Limits posed by specific work issues, such as working with machinery or driving license restrictions, can affect employment status in PwE [43, 44].

To summarize, we can refer to Gloag's paper in which he highlighted three aspects impairing job position in PwE: the hazards posed by specific jobs in case of seizures; anxiety and prejudice on the side of employers and fellow workers; behavioral and mental abnormalities [3]. Geerlings has also drawn a specific predicting risk profile score made up of unsupportive family environment, lower intelligence, and higher seizure frequency and ongoing seizures [32].

### 3.3 Underemployment and Lower Salaries

Researchers have shown evidence of disadvantages for PwE in the workplace, even when a higher rate of unemployment is not reported. PwE earned lower salaries [9, 33] and were underemployed compared with the general population [45, 46, 47]. These issues may derive from lower educational levels [21], employers' attitudes, misconceptions, and self‐denial.

### 3.4 Factors Favoring Employment

Factors opposed to those described previously facilitate employment. The most reported factor favoring employment is seizure remission or good seizure control [4, 23, 28, 30, 48].

People with normal intelligence are prone to get a better education and, consequently, better employment [25, 28, 49]. Focusing on specific functions, Partanen demonstrated that better working memory and executive functions were associated with improvement in employment status in surgical patients [50].

A higher educational level allows PwE to reach a more suitable job [20, 24, 31, 51, 52].

Experiences from Ireland, UK, and Belgium demonstrated that training and job counseling favored the employment of PwE more than other elements [30, 49, 53].

Having a favorable atmosphere at work, strong relationships, and a positive individual perception increase the chances of being employed [4, 51].

Although social aspects are less explored, we can assume that emotional stability (including having no psychiatric comorbidities) and proper social skills positively contribute to PwE's employment status [36].

As declared before, employment status is strictly connected to seizure control and seizure freedom [24, 26, 30, 37, 41, 49, 54]. Other clinical factors, such as being treated with a lower number of antiseizure medications, can contribute to a good employment situation [55]. When considering long‐term outcomes, surgery has a modest but significant influence on employment status in surgical candidates [26, 56, 57]. Finally, having a driving license also contributes to independence and, hence, improves the employment situation in PwE [44].

Considering external factors, apart from employers' attitudes, the promulgation of laws favoring inclusion of PwE in the workplace represents one of the most relevant determinants supporting employment, as proven in Germany [18].

### 3.5 Long‐Term Outcomes and Specific Conditions

When analyzing specific conditions:

#### 3.5.1 Non‐Surgical Patients

– In Sweden, Olsson analyzed long‐term outcomes in people with absence epilepsy. In about 74% of cases, epilepsy impaired social aspects, such as schooling, occupation, and relationships, regardless of seizure control [46].
– Peña demonstrated worse outcomes in people with higher scores of anxiety and depression in a population of people with refractory epilepsy [40].
– Two studies focused on the outcomes of people with childhood‐onset epilepsy. Geerts evidenced lower job attainment of people with childhood‐onset epilepsy, despite remission [21]. Sillanpaa demonstrated better employment outcomes in people with normal intelligence, onset of epilepsy greater than 6 years old, good vocational education, uninterrupted remission, and no history of status epilepticus [28].
– Schneider‐Von Podewils drew attention to the special situation of Janz syndrome (now defined as Juvenile myoclonic epilepsy), specifying that a higher number of GTC seizures was associated with a worse outcome [34].

#### 3.5.2 Surgical Patients

– Reihnoldson proved that good surgical outcomes in children depend on normal intelligence, seizure freedom, and higher age at surgery: people with these features show higher employment rates [25].
– Partanen in Finland revealed that normal cognitive functions are correlated with better social outcomes after surgery [50].
– Edelvik evaluated long‐term outcomes after resective surgery: younger people, seizure‐free patients, and people employed/studying at baseline develop better employment outcomes [24].
– In cases susceptible to surgical treatment, surgery seems to improve employment status in PwE: we can reach this conclusion by comparing outcomes in people who undergo surgery [56] and people who do not [57].
– Extratemporal surgery leads to a better quality of life and, consequently, a better psychosocial outcome, especially in people gaining seizure freedom [26].
– Concerning long‐term (> 10 years) psychosocial and socioeconomic outcomes of pediatric epilepsy surgery, Hoppe demonstrated that effective surgery leads PwE to better social outcomes. Likewise, comorbid disabilities impair employment attainment, whereas seizure freedom improves it. They also underlined the difference in the employment rate for PwE who had unilobar surgery (76%) and PwE who had multilobar surgery (10%) [41].
– Finally, Dupont focused on surgery in medial temporal lobe epilepsy, demonstrating the intrinsic positive value of surgery on employment status [56].

### 3.6 Disclosure Issues

Though it was not our primary aim, we could not leave unmentioned papers that shed light on such a controversial issue as disclosure. Most PwE fear discrimination, stigmatization, and negative consequences (such as being fired, demoted or unemployed) when revealing their condition. They frequently conceal their disorder, especially if well controlled [4, 15, 39]. In different studies, about one‐half or one‐third of the population examined did not disclose their condition [14, 23, 51]. Lassouw evidenced that in a Dutch working population, 77% of respondents disclosed their condition, although they refused to answer questions about the impact of epilepsy on their job [9].

### 3.7 Employment Programs and Interventions for PwE in Europe

In Europe we found a report of only a few experiences with inclusion programs. In 1992, Carroll demonstrated the usefulness of a rehabilitation program in the Irish population: 58% of the considered population had found employment at the end of the program. Furthermore, about 66% of the surveyed population thought that programs are useful to develop self‐confidence and social skills [53]. Similarly, in Sweden, Wedlund demonstrated improvement in work and education participation in 38 among 124 PwE involved in a rehabilitation program [58].

## 4 Discussion

Our systematic review on employment of PwE resulted in both lights and shadows. Defining the real unemployment rate among PwE remains difficult, as reported rates vary widely—from similar to two or three times higher—depending on the countries and years examined. In the last two decades, a slight prevalence of articles reporting similar employment rates (five articles reporting similar rates versus three papers reporting lower rates of employment) is evident, maybe because of increased awareness about epilepsy.

Conflicting results could depend on different ways to consider or define unemployment, with variations found when including or excluding housewives, students, and retired people.

Yet a clear disadvantage emerges from all European studies [47, 51, 56]. PwE frequently face underemployment or disadvantages like lower salaries, because of varied factors: lower educational attainment, lack of protective policies, stigma, misconceptions, and discriminatory employers' attitudes [9, 21, 33, 45, 46, 47].

Among clinical factors, unsurprisingly, seizure control plays a key role and represents the most reported aspect favoring a suitable job position for PwE. Whether achieved through medications or surgery, it enables better outcomes in all social domains, including job, marriage, and quality of life. Seizure‐free people can lead a “normal” life without limitations for driving, cognitive functioning, marriage, and employment [36], especially if they have good social skills [36]. They are able to achieve higher educational levels and fulfilling careers. Thus, for neurologists, healthcare professionals, and PwE alike, achieving seizure freedom is a matter of utmost importance, as it promotes meaningful inclusion in work and society.

Stigma still represents a significant barrier to employment for PwE. Perceived stigma and discriminatory attitudes (both from employers and families) can severely limit job opportunities and quality of life of PwE [59]. Misconceptions and restrictions contribute to stigma, leading PwE to conceal their condition, often out of fear of discrimination or job loss. This results in a reduction in ambition, emotional stability, and job attainment. The International Bureau for Epilepsy (IBE) and the International League Against Epilepsy (ILAE) are actively working to fight stigma and eliminate unjustifiable restrictions on PwE.

Indeed, educational and rehabilitation programs are other relevant instruments to truly achieve the desired position, especially in PwE with normal intelligence and the absence of psychiatric comorbidities [53, 58].

This review also devotes special attention to one of the challenges PwE face in obtaining a job: disclosure. Many PwE tend to not disclose their condition, unless necessary, fearing underemployment or dismissal due to ignorant prejudices. Actually, most PwE can work safely, without experiencing seizures and posing risks for themselves or colleagues. A supportive and inclusive workplace environment can encourage disclosure and foster trust between employers and employees. The IBE's 2007 leaflet highlights the importance of creating such environments to fight stigma and promote inclusion [60].

To address these challenges, the EpilepsyPOWER project aims to dispel myths and improve workplace inclusion for PwE. Future steps for the project include evaluating employer knowledge of epilepsy and work conditions for PwE in Europe through anonymous surveys, addressing knowledge gaps identified in this review. Additionally, the creation of collaborative laboratories and educational modules for employers will promote inclusion and eliminate unfair restrictions for PwE in the workplace.

To sum up, we can depict the key elements for PwE to obtain an optimal job position: seizure control, normal cognitive functioning, higher educational levels, and a supportive atmosphere (both at work and in the family).

### 4.1 Strengths and Limitations

Our review seems to be comprehensive and extensive, describing the unemployment situation in Europe over the last 60 years, including longitudinal studies about specific conditions. The wide extension of years and conditions considered makes this review noteworthy, laying the background for future studies in the social field.

We have focused on European literature as this review is a deliverable of the EpilepsyPOWER European Project Erasmus + (project 2021‐1‐IT02‐KA220‐ADU‐000028349).

Main limitations are variability among reported rates of employment/unemployment, different sample dimensions, different times of observation (longitudinal vs. transversal studies), and methodologies of reported studies. Another limitation is the heterogeneity of considered populations as employed (e.g., part‐time) and unemployed (e.g., housewives).

Most of the reviewed papers used surveys, so they could be affected by compilation biases, and finally, not all the papers considered the comparison with the general population.

## 5 Conclusion

This review enlightens the difficulties faced every day by PwE in workplaces. Although rates of unemployment are not uniformly reported, a clear situation of disadvantage rises for PwE in Europe. Reasons for unemployment range from seizure control to discriminatory employers' attitudes. Indeed, spreading awareness and knowledge about epilepsy among employers and caregivers could foster inclusion in workplaces, fighting stigma and ignorance.

Providing PwE the right tools to choose the most suitable job and information about their disorder could help them to obtain the appropriate position.

Finally, a good seizure control could help PwE get and retain a job, leading them to independence, better self‐esteem, and social integration.

## Author Contributions

**F. Narducci:** conceptualization, writing – original draft, writing – review and editing, investigation, formal analysis, methodology. **G. Lippa:** investigation. **G. Baker:** data curation. **D. Walsh:** project administration. **F. Sofia:** project administration. **N. Casalino:** funding acquisition, project administration. **F. Pigni:** funding acquisition, project administration. **S. Louissi:** investigation. **I. Philipova:** visualization. **S. Duttenhöfer:** resources. **L. Ricci:** visualization. **B. Sancetta:** visualization. **J. Lanzone:** software, methodology. **M. Tombini:** supervision. **V. Di Lazzaro:** supervision. **G. Assenza:** methodology, validation, supervision.

## Conflicts of Interest

The authors declare no conflicts of interest.

## Data Availability

The data that support the findings of this study are available from the corresponding author upon reasonable request.

## References

[1] R. E. D. Bautista , D. Shapovalov , F. Saada , and M. A. Pizzi , “The Societal Integration of Individuals With Epilepsy: Perspectives for the 21st Century,” Epilepsy and Behavior 35 (2014): 42–49, 10.1016/j.yebeh.2014.04.006. 24798409

[2] E. Beghi , “The Epidemiology of Epilepsy,” Neuroepidemiology 54 (2020): 185–191, 10.1159/000503831. 31852003

[3] D. Gloag , “Epilepsy and Employment,” British Medical Journal (Clinical Research Ed.) 291, no. 6487 (1985): 2–3, 10.1136/BMJ.291.6487.2. 3926043 PMC1416167

[4] J. A. Collings and B. Chappell , “Correlates of Employment History and Employability in a British Epilepsy Sample,” Seizure 3, no. 4 (1994): 255–262, 10.1016/S1059-1311(05)80172-4. 7894835

[5] A. Jacoby , J. Gorry , and G. A. Baker , “Employers' Attitudes to Employment of People With Epilepsy: Still the Same Old Story?,” Epilepsia 46 (2005): 1978–1987. 16393165 10.1111/j.1528-1167.2005.00345.x

[6] M. Jahoda , Employment and Unemployment: A Social Psychological Analysis (Cambridge University Press, 1982).

[7] R. S. Taylor , J. W. Sander , R. J. Taylor , and G. A. Baker , “Predictors of Health‐Related Quality of Life and Costs in Adults With Epilepsy: A Systematic Review,” Epilepsia 52, no. 12 (2011): 2168–2180, 10.1111/J.1528-1167.2011.03213.X. 21883177

[8] T. Nishida , K. Terada , H. Ikeda , and Y. Inoue , “Seizures, Accidental Injuries at Work, and Reasons for Resignation in People With Epilepsy,” Epilepsy & Behavior 111 (2020): 107237, 10.1016/J.YEBEH.2020.107237. 32575014

[9] G. Lassouw , P. Lefferst , M. De Krom , and J. Troost , “Epilepsy in a Dutch Working Population: Are Employees Diagnosed With Epilepsy Disadvantaged?,” Seizure 6, no. 2 (1997): 95–98, 10.1016/S1059-1311(97)80061-1. 9153720

[10] V. M. J. Smeets , B. A. G. van Lierop , J. P. G. Vanhoutvin , A. P. Aldenkamp , and F. J. N. Nijhuis , “Epilepsy and Employment: Literature Review,” Epilepsy and Behavior 10, no. 3 (2007): 354–362, 10.1016/j.yebeh.2007.02.006. 17369102

[11] M. C. M. Wo , K. S. Lim , W. Y. Choo , and C. T. Tan , “Employability in People With Epilepsy: A Systematic Review,” Epilepsy Research 116 (2015): 67–78, 10.1016/j.eplepsyres.2015.06.016. 26354169

[12] M. Ouzzani , H. Hammady , Z. Fedorowicz , and A. Elmagarmid , “Rayyan‐a Web and Mobile App for Systematic Reviews,” Systematic Reviews 5 (2016): 210, 10.1186/s13643-016-0384-4. 27919275 PMC5139140

[13] A. Liberati , D. G. Altman , J. Tetzlaff , et al., “The PRISMA Statement for Reporting Systematic Reviews and Meta‐Analyses of Studies That Evaluate Health Care Interventions: Explanation and Elaboration,” Journal of Clinical Epidemiology 62, no. 10 (2009): e1–e34, 10.1016/J.JCLINEPI.2009.06.006. 19631507

[14] G. Scambler and A. Hopkins , “Social Class, Epileptic Activity, and Disadvantage at Work,” Journal of Epidemiology & Community Health 34, no. 2 (1980): 129–133, 10.1136/jech.34.2.129. 7400725 PMC1052057

[15] A. Jacoby , “Epilepsy and the Quality of Everyday Life Findings From A Study of People With Well‐Controlled Epilepsy,” Social Science & Medicine 34, no. 6 (1992): 657–666, 10.1016/0277-9536(92)90193-t. 1574733

[16] A. Jacoby , “Impact of Epilepsy on Employment Status: Findings From a UK Study of People With Well‐Controlled Epilepsy,” Epilepsy Research 21, no. 2 (1995): 125–132, 10.1016/0920-1211(95)00013-z. 7588587

[17] N. Callaghan , M. Crowley , and T. Goggin , “Epilepsy and Employment, Marital, Education and Social Status,” Irish Medical Journal 85 (1992): 17–19. 1568840

[18] A. Korchounov , T. Tabatadze , D. Spivak , and W. Rössy , “Epilepsy‐Related Employment Prevalence and Retirement Incidence in the German Working Population: 1994–2009,” Epilepsy and Behavior 23, no. 2 (2012): 162–167, 10.1016/j.yebeh.2011.09.017. 22236573

[19] M. Herodes , A. Õun , S. Haldre , and A. E. Kaasik , “Epilepsy in Estonia: A Quality‐of‐Life Study,” Epilepsia 42, no. 8 (2001): 1061–1073, 10.1046/j.1528-1157.2001.0420081061.x. 11554894

[20] M. Rätsepp , A. Õun , S. Haldre , and A. E. Kaasik , “Felt Stigma and Impact of Epilepsy on Employment Status Among Estonian People: Exploratory Study,” Seizure 9, no. 6 (2000): 394–401, 10.1053/seiz.2000.0439. 10985995

[21] A. Geerts , O. Brouwer , C. Van Donselaar , et al., “Health Perception and Socioeconomic Status Following Childhood‐Onset Epilepsy: The Dutch Study of Epilepsy in Childhood,” Epilepsia 52, no. 12 (2011): 2192–2202, 10.1111/j.1528-1167.2011.03294.x. 22004073

[22] A. Marinas , E. Elices , A. Gil‐Nagel , et al., “Socio‐Occupational and Employment Profile of Patients With Epilepsy,” Epilepsy and Behavior 21, no. 3 (2011): 223–227, 10.1016/j.yebeh.2011.01.025. 21620775

[23] B. Majkowska‐Zwolińska , J. Jedrzejczak , and K. Owczarek , “Employment in People With Epilepsy From the Perspectives of Patients, Neurologists, and the General Population,” Epilepsy and Behavior 25, no. 4 (2012): 489–494, 10.1016/j.yebeh.2012.10.001. 23153712

[24] A. Edelvik , R. Flink , and K. Malmgren , “Prospective and Longitudinal Long‐Term Employment Outcomes After Resective Epilepsy Surgery From the Department of Clinical Neuroscience and Rehabilitation,” Neurology 85, no. 17 (2015): 1482–1490. 26408490 10.1212/WNL.0000000000002069 PMC4631069

[25] J. Reinholdson , I. Olsson , A. Edelvik Tranberg , and K. Malmgren , “Long‐Term Employment Outcomes After Epilepsy Surgery in Childhood,” Neurology 94, no. 2 (2020): E205–E216, 10.1212/WNL.0000000000008681. 31796526 PMC6988983

[26] K. Walther , M. Dogan Onugoren , M. Buchfelder , et al., “Psychosocial Outcome in Epilepsy After Extratemporal Surgery,” Epilepsy and Behavior 81 (2018): 94–100, 10.1016/j.yebeh.2018.01.038. 29454606

[27] P. Jennum , N. M. M. Debes , R. Ibsen , and J. Kjellberg , “Long‐Term Employment, Education, and Healthcare Costs of Childhood and Adolescent Onset of Epilepsy,” Epilepsy and Behavior 114 (2021): 107256, 10.1016/j.yebeh.2020.107256. 32622728

[28] M. Sillanpää and D. Schmidt , “Long‐Term Employment of Adults With Childhood‐Onset Epilepsy: A Prospective Population‐Based Study,” Epilepsia 51, no. 6 (2010): 1053–1060, 10.1111/j.1528-1167.2009.02505.x. 20163443

[29] A. Jacoby , G. A. Baker , N. Steen , P. Potts , and W. Chadwick , “The Clinical Course of Epilepsy and Its Psychosocial Correlates: Findings From a U.K. Community Study,” Epilepsia 37, no. 2 (1996): 148–161, 10.1111/j.1528-1157.1996.tb00006.x. 8635425

[30] J. E. Chaplin , A. Wester , and T. Tomson , “Factors Associated With the Employment Problems of People With Established Epilepsy,” Seizure 7, no. 4 (1998): 299–303, 10.1016/S1059-1311(98)80022-8. 9733405

[31] M. Herodes , A. Õun , S. Haldre , and A. E. Kaasik , “Epilepsy in Estonia: A Quality‐of‐Life Study.” 10.1046/j.1528-1157.2001.0420081061.x 11554894

[32] R. P. J. Geerlings , A. P. Aldenkamp , L. M. C. Gottmer‐Welschen , et al., “Developing From Child to Adult: Risk Factors for Poor Psychosocial Outcome in Adolescents and Young Adults With Epilepsy,” Epilepsy and Behavior 51 (2015): 182–190, 10.1016/j.yebeh.2015.07.035. 26291772

[33] P. Jennum , J. Gyllenborg , and J. Kjellberg , “The Social and Economic Consequences of Epilepsy: A Controlled National Study,” Epilepsia 52, no. 5 (2011): 949–956, 10.1111/j.1528-1167.2010.02946.x. 21275976

[34] F. Schneider‐Von Podewils , C. Gasse , J. Geithner , et al., “Clinical Predictors of the Long‐Term Social Outcome and Quality of Life in Juvenile Myoclonic Epilepsy: 20–65 Years of Follow‐Up,” Epilepsia 55, no. 2 (2014): 322–330, 10.1111/epi.12491. 24417603

[35] N. Gordon and S. Russell , “The Problem of Unemployment Among Epileptics,” Journal of Mental Science 104, no. 434 (1958): 103–114, 10.1192/BJP.104.434.103. 13514449

[36] P. J. Thompson and J. Oxley , “Socioeconomic Accompaniments of Severe Epilepsy,” Epilepsia 29 (1988): S9–S18, 10.1111/j.1528-1157.1988.tb05791.x. 3391156

[37] D. L. Mclellan , “Epilepsy and Employment,” Occupational Medicine 37 (1987): 94–99. 10.1093/occmed/37.1.94 3431107

[38] M. G , L. GL , M. J , et al., “Clinical Factors Associated With Work Disability in Epilepsy: A Cross‐Sectional Study at a Tertiary Referral Hospital,” Epilepsy and Behavior 124 (2021): 108310, 10.1016/j.yebeh.2021.108310. 34530247

[39] D. A. Pond and B. H. Bidwell , “A Survey of Epilepsy in Fourteen General Practices. II. Social and Psychological Aspects,” Epilepsia 1, no. 1–5 (1960): 285–299, 10.1111/J.1528-1157.1959.TB04266.X. 14433986

[40] P. Peña , J. Sancho , M. Rufo , S. Martínez , and J. Rejas , “Driving Cost Factors in Adult Outpatients With Refractory Epilepsy: A Daily Clinical Practice in Clinics of Neurology in Spain,” Epilepsy Research 83, no. 2–3 (2009): 133–143, 10.1016/j.eplepsyres.2008.10.004. 19095410

[41] C. Hoppe , K. Beeres , J. A. Witt , R. Sassen , and C. Helmstaedter , “How Are They Doing as Adults? Psychosocial and Socioeconomic Outcomes 11–30 Years After Pediatric Epilepsy Surgery,” Epilepsia Open 8, no. 3 (2023): 797–810, 10.1002/EPI4.12736. 37003960 PMC10472367

[42] A. Haag , A. Strzelczyk , S. Bauer , S. Kühne , H. M. Hamer , and F. Rosenow , “Quality of Life and Employment Status Are Correlated With Antiepileptic Monotherapy Versus Polytherapy and Not With Use of “Newer” Versus “Classic” Drugs: Results of the “Compliant 2006” Survey in 907 Patients,” Epilepsy and Behavior 19, no. 4 (2010): 618–622, 10.1016/j.yebeh.2010.09.037. 21115406

[43] R. D. C. Elwes , J. Marshall , A. Beattie , P. K. Newman , and T. A. Beattie , “Epilepsy and Employment. A Community Based Survey in an Area of High Unemployment,” Journal of Neurology, Neurosurgery & Psychiatry 54, no. 3 (1991): 200–203, 10.1136/jnnp.54.3.200. 2030345 PMC1014384

[44] J. Schulz , A. Beicher , G. Mayer , et al., “Counseling and Social Work for Persons With Epilepsy: Observational Study on Demand and Issues in Hessen, Germany,” Epilepsy and Behavior 28, no. 3 (2013): 358–362, 10.1016/j.yebeh.2013.05.027. 23832132

[45] N. Callaghan , “Epilepsy and Employment, Marital, Education and Social Status,” Irish Medical Journal 1 (1992): 17–19. 1568840

[46] I. Olsson and G. Campenhausen , “Social Adjustment in Young Adults With Absence Epilepsies,” Epilepsia 34, no. 5 (1993): 846–851, 10.1111/j.1528-1157.1993.tb02101.x. 8404736

[47] D. Shackleton , D. Kasteleijn‐Nolst Trenité , A. de Craen , J. Vandenbroucke , and R. Westendorp , “Living With Epilepsy Long‐Term Prognosis and Psychosocial Outcomes,” Neurology 61, no. 1 (2003): 64–70, 10.1212/01.wnl.0000073543.63457.0a. 12847158

[48] E. Beghi , C. M. Cornaggia , W. A. Hauser , et al., “Social Aspects of Epilepsy in the Adult in Seven European Countries,” Epilepsia 41, no. 8 (2000): 998–1004, 10.1111/J.1528-1157.2000.TB00285.X. 10961627

[49] L. Sorel , “The Epileptic Worker in the Construction Industry,” Epilepsia 13, no. 1 (1972): 57–62, 10.1111/J.1528-1157.1972.TB04550.X. 4501899

[50] E. Partanen , S. Laari , O. Kantele , L. Kämppi , and T. Nybo , “Associations Between Cognition and Employment Outcomes After Epilepsy Surgery,” Epilepsy and Behavior 131 (2022): 108709, 10.1016/j.yebeh.2022.108709. 35526464

[51] J. J. Graham , “Employment of Epileptics,” Lancet 286, no. 7410 (1965): 486–489, 10.1016/S0140-6736(65)91441-8. 14337839

[52] A. Koponen , U. Seppälä , K. Eriksson , et al., “Social Functioning and Psychological Well‐Being of 347 Young Adults With Epilepsy Only—Population‐Based, Controlled Study From Finland,” Epilepsia 48, no. 5 (2007): 907–912, 10.1111/j.1528-1167.2007.01017.x. 17430406

[53] D. Carroll , “Employment Among Young People With Epilepsy,” Seizure 1, no. 2 (1992): 127–131. 1344327 10.1016/1059-1311(92)90010-x

[54] R. Schulz , H. O. Lüders , M. Hoppe , et al., “Lack of Aura Experience Correlates With Bitemporal Dysfunction in Mesial Temporal Lobe Epilepsy,” Epilepsy Research 43, no. 3 (2001): 201–210, 10.1016/S0920-1211(00)00195-9. 11248532

[55] H. M. De Boer , “Overview and Perspectives of Employment in People With Epilepsy,” Epilepsia 46 (2005): 52–54. 10.1111/j.0013-9580.2005.461016.x 15816982

[56] S. Dupont , M. L. Tanguy , S. Clemenceau , C. Adam , P. Hazemann , and M. Baulac , “Long‐Term Prognosis and Psychosocial Outcomes After Surgery for MTLE,” Epilepsia 47, no. 12 (2006): 2115–2124, 10.1111/j.1528-1167.2006.00852.x. 17201711

[57] M. Carreño , A. Donaire , and R. Sánchez‐Carpintero , “Cognitive Disorders Associated With Epilepsy: Diagnosis and Treatment,” Neurologist 14, no. 6 (2008): 26–34, 10.1097/01.nrl.0000340789.15295.8f. 19225368

[58] E. W. Wedlund , L. Nilsson , A. Erdner , and T. Tomson , “Long‐Term Follow‐Up After Comprehensive Rehabilitation of Persons With Epilepsy, With Emphasis on Participation in Employment or Education,” Epilepsy and Behavior 25, no. 2 (2012): 219–223, 10.1016/j.yebeh.2012.06.029. 23032136

[59] M. Tombini , G. Assenza , L. Quintiliani , L. Ricci , J. Lanzone , and V. Di Lazzaro , “Epilepsy and Quality of Life: What Does Really Matter?,” Neurological Sciences 42, no. 9 (2021): 3757–3765, 10.1007/S10072-020-04990-6. 33449244

[60] https://www.ibe‐epilepsy.org/downloads/Employment%20Guidelines.pdf.

